# Spatiotemporal-based automated inundation mapping of Ramsar wetlands using Google Earth Engine

**DOI:** 10.1038/s41598-023-43910-4

**Published:** 2023-10-13

**Authors:** Manish Kumar Goyal, Shivukumar Rakkasagi, Soumya Shaga, Tian C. Zhang, Rao Y. Surampalli, Saket Dubey

**Affiliations:** 1 Department of Civil Engineering, Indian Institute of Technology, Indore, India; 2 Department of Civil and Environmental Engineering, University of Nebraska, Lincoln, NE USA; 3 Global Institute for Energy, Environment, and Sustainability, Lenexa, KS USA; 4 School of Infrastructure, Indian Institute of Technology, Bhubaneswar, India

**Keywords:** Wetlands ecology, Hydrology

## Abstract

Wetlands are one of the most critical components of an ecosystem, supporting many ecological niches and a rich diversity of flora and fauna. The ecological significance of these sites makes it imperative to study the changes in their inundation extent and propose necessary measures for their conservation. This study analyzes all 64 Ramsar sites in China based on their inundation patterns using Landsat imagery from 1991 to 2020. Annual composites were generated using the short-wave infrared thresholding technique from June to September to create inundation maps. The analysis was carried out on each Ramsar site individually to account for its typical behavior due to regional geographical and climatic conditions. The results of the inundation analysis for each site were subjected to the Mann–Kendall test to determine their trends. The analysis showed that 8 sites exhibited a significantly decreasing trend, while 14 sites displayed a significantly increasing trend. The accuracy of the analysis ranged from a minimum of 72.0% for Hubei Wang Lake to a maximum of 98.0% for Zhangye Heihe Wetland National Nature Reserve. The average overall accuracy of the sites was found to be 90.0%. The findings emphasize the necessity for conservation strategies and policies for Ramsar sites.

## Introduction

Despite covering only about 2.6% of the earth’s land area, wetlands are vital to the hydrological cycle and play a significant role in regulating water flow and quality^1^. Moreover, wetlands are responsible for the production of over 20% of the earth's organic carbon^2^, making them an essential source of nutrients and energy for many aquatic and terrestrial ecosystems^3^. The availability of adequate food and water makes them the best place for diverse species forms^4, 5^. Wetlands provide a wide range of vital ecosystem services, such as purifying water, controlling floods, conserving biodiversity, supplying food, and sequestering carbon^6^. Unfortunately, as one of the most vulnerable ecosystems, wetlands have suffered significant losses and degradation worldwide due to climate change and human activities^7–9^. As wetlands have a close relationship with the climate, any changes in their behavior reflect the changing climatic conditions and vice versa^10, 11^.

The Ramsar Convention, also known as the Wetlands Convention, is an international treaty signed on February 2, 1971, in the city of Ramsar, Iran^12^. Its objective is to conserve and sustainably use wetlands, which are designated as Ramsar sites (Please refer to S1 in the Supplementary Information to understand the criteria adopted for identification of wetland as a Ramsar site). The convention provides a framework for the protection and responsible use of wetlands^11^. The mission of the convention is to achieve sustainable development worldwide by conserving and wisely using wetlands through local and national actions and international cooperation^13^. Implementation of the Ramsar Strategic Plan contributes to the achievement of the Sustainable Development Goals (SDGs)^14^. The Ramsar Conventions' fourth strategic plan (2016–2024) identifies addressing the drivers of wetland loss and degradation, effectively conserving and managing the Ramsar site network, wisely using all wetlands, and enhancing implementation as four overarching goals^13^. Most of the proposed SDGs are relevant in some way or another to wetlands, but the following are of particular importance: wetlands ensure fresh water, help replenish ground aquifers, and purify and filter harmful waste from water (Goal 6 of SDG)^14^. Rice grown in wetland paddies is the staple diet of nearly three billion people (Goal 2 of SDG)^13^. They also help reduce drought and contribute to the land formation and coastal zone stability by regulating sediment transport (Goal 11 of SDG)^15, 16^. Wetlands act as carbon sinks and coastal wetlands reduce the impact of rising sea levels, acting as storm surge buffers and providing erosion control (Goal 13 of SDG). Without wetlands, the water, carbon, and nutrient cycles would be significantly altered (Goal 14 and 15 of SDG)^13^.

Mainland China (China), with the fourth-largest wetland coverage in the world, has wetlands that cover 5% of the country's territorial area^17^. The country is home to 64 Ramsar sites, covering over 7.3 million hectares (73,000 square kilometres) of area^18^. Wetlands in China, in spite of their international importance, have been rapidly declining^19, 20^. Recognizing this issue, restoration has become a major focus of the Ramsar Convention. Numerous studies have explored wetland changes and the factors driving them^17, 21^. For example, it has been estimated that China's total wetland area decreased by over 50,000 km^2^ between 1990 and 2000, and that 33% of the country's wetlands were lost between 1978 and 2008^22, 23^. The loss of vegetated wetlands in China between 1990 and 2010 was primarily due to agricultural expansion^24^.

Inundation maps are important for analyzing the historical and present status of wetlands, forecasting future changes, and understanding the impact of climate change, natural phenomena, and human resources^25–27^. They are also useful for wetland management plans and biodiversity research^28^. However, wetland change detection and delineation that are comprehensive and timely are impossible to obtain using only standard in-situ methods of data collection^29^. Satellite images can be used to prepare inundation maps for extensive or inaccessible regions or when the workforce is limited^30^. However, inundation mapping is complicated due to the regular and non-uniform variations in the extent of inundation. Automating inundation mapping using machine learning algorithms such as Support Vector Machine (SVM), Random Forest (RF) classifiers, etc. can be a better way to comprehend wetlands^31^. Recent developments in machine learning techniques and cloud-computing platforms such as Google Earth Engine (GEE) have made complete, large-scale wetland delineation maps possible^29, 32–34^. GEE provides access to remotely sensed satellite data and significant computational capability for processing and analyzing satellite images^34, 35^. Remote monitoring of wetlands using satellite data has become a successful way for long-term, systematic wetland mapping^36, 37^.

In this study, we utilized Landsat data of 64 Ramsar sites in China to create a lengthy sequence of peak inundation maps for the delta, with a medium spatial resolution of 30 m. We established an automated version of a previous technique that relies on SWIR band thresholding in GEE, a cloud-based tool for geospatial analysis. Results indicate that the SWIR thresholding method is dependable. The inundation maps and GEE code are now available for stakeholders, land managers, and academics to use and modify.

## Study area

**Figure 1:**
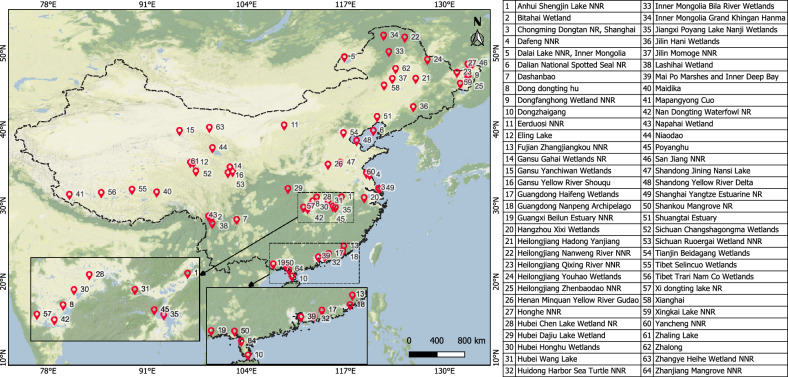
Location map of Ramsar wetland sites in mainland China. The table represents names of the wetlands (NNR: National Nature Reserve and NR: Nature Reserve). This figure is created using QGIS 3.30.1 (Quantum Geographic Information System; https://download.qgis.org/downloads/) and the background shows the base-map of Stamen terrain from HCMGIS Plugin.

China became a member of the Ramsar Convention in 1992 and has since designated 64 Ramsar sites as of 2021, located in 24 provincial-level regions (Fig. 1). Heilongjiang has the most sites with 10, followed by Gansu, Guangdong, Hubei, Yunnan, Tibet, and Inner Mongolia with four sites each. These wetlands include all types defined by the Ramsar Convention and have a carbon sink capacity of more than 1.71 million metric tons per year^38^. The Ramsar sites in China cover various natural wetland types such as swamps, marshes, lakes, rivers, mangroves, tidal flats, estuaries, and shallow marine water. Of these, 17 receive more than 1500 mm of mean annual precipitation; 25 and 23 sites were found in the humid subtropical and continental climate regions, respectively. The majority of the sites are located in low-altitude regions, with 35 sites at an elevation of less than 250 m, and most have a mean temperature of less than 20 °C but a maximum temperature greater than 30 °C^38, 39^. Please refer to Table S3a and b in the Supplementary Information for the details such as area, latitude, longitude, wetland type, selection criteria, maximum, and minimum elevation of each Ramsar site of China.

## Results and discussion

### Inundation maps

**Figure 2:**
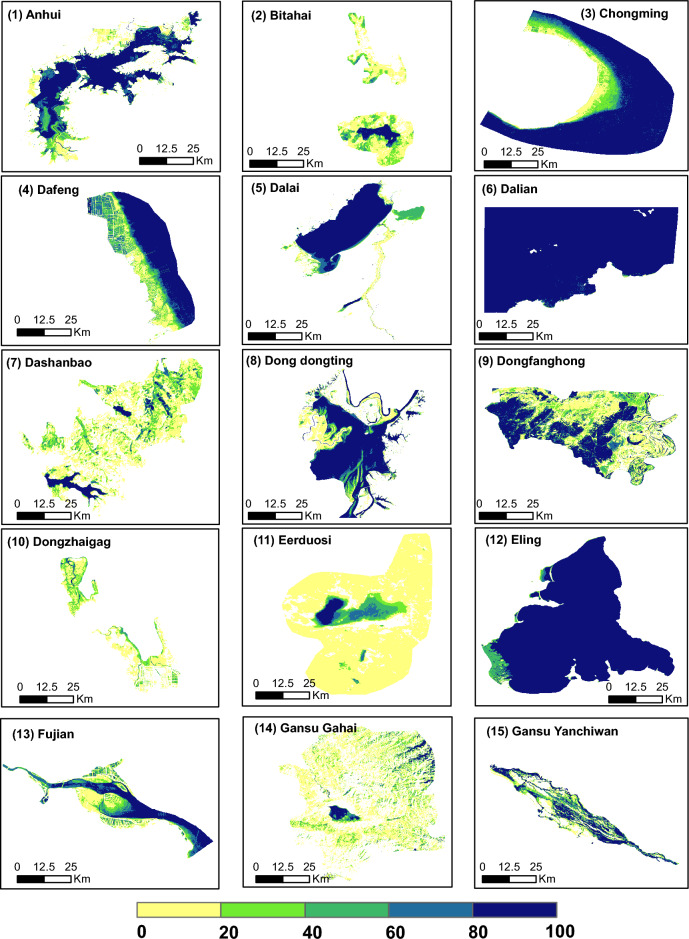
Inundation maps represent the number of years each pixel was inundated during the period 1991–2020 (June to September). This figure represents the first 15 out of 64 Ramsar sites. This figure is created using QGIS 3.30.1 (Quantum Geographic Information System; https://download.qgis.org/downloads/).

This paper tried to evaluate the three-decade inundation pattern in China Ramsar sites. It cleared the way for a deeper comprehension of surface water dynamics and the ability to predict upcoming trends. The total area of shapefiles used in this analysis for China over a 30-year period was 73,269.52 km^2^. The thresholding technique produced inundation maps that displayed annual fluctuation at each site. Each site displayed a distinctive pattern of variation, in varying degrees, which was explicable by topographical and climatological factors^27, 40^. Figures 2 display the inundation frequency maps for 15 Ramsar sites (the remaining maps of 49 Ramsar sites are included in the Supplementary Information; Fig. S2a–d). On these maps, 100% (dark blue) pixels denote areas that have experienced flooding in all the timesteps that are available, whereas 0% (yellow) pixels denote areas that have experienced flooding in none of the timesteps and are, therefore, can be considered permanently dry^41^.

The wetlands were divided into five classes as follows to effectively analyze the data related to the extent of variation in the inundation and draw conclusions.

**Figure 3:**
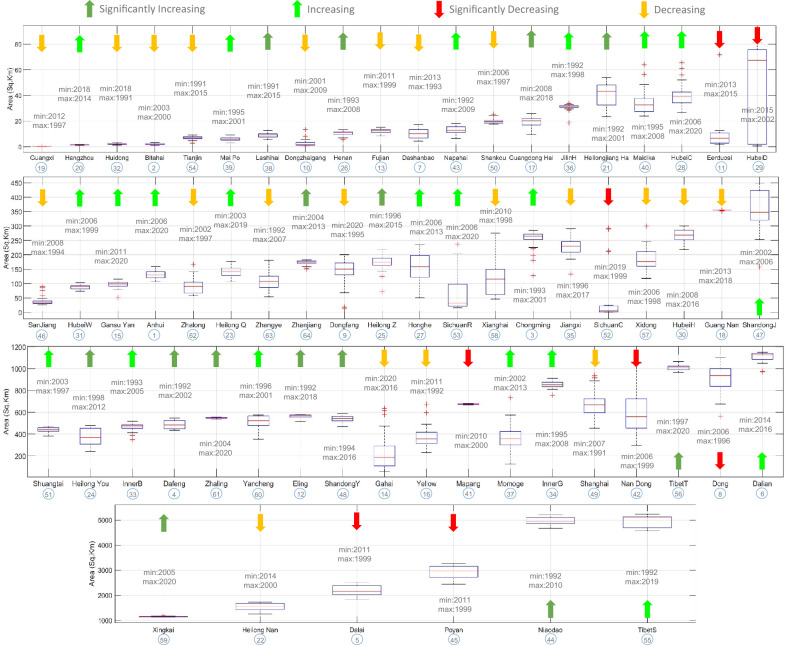
Boxplot representing the minimum, 1st quartile, mean, 3rd quartile, and maximum inundated areas for each Ramsar site. The arrows on each site represent the trend of the wetland during the period 1991–2020 using the Mann–Kendall test. Also, the year of occurrence of minimum and maximum inundation is mentioned for each site. This figure is created using MATLAB R2022a (https://www.mathworks.com/products/new_products/release2022a.html).

- High-altitude wetlands are those wetlands whose elevation is greater than 4000 m. Nine wetlands are in this category, namely Bitahai Wetland, Gansu Yellow River Shouqu Wetlands, Zhaling Lake, Eling Lake, Tibet Trari Nam Co Wetlands Sichuan Changshagongma Wetlands, Maidika, and Mapangyong Cuo. Throughout the time series, i.e., 30 years, eight showed variation such that their maximum inundation extent was less than three times their minimum extent. However, Sichuan Changshagongma Wetlands exceptionally have their maximum inundation extent extremely greater than three times (~ 204.32 times) their minimum inundation extent (Fig. 3).
- Coastal wetlands are wetlands that are directly connected to the sea. As a result, their water level and hence the inundation extent was controlled by the sea level. There are 16 wetlands that were found under this category namely, Chongming Dongtan Nature Reserve, Dalian National Spotted Seal Nature Reserve, Dafeng National Nature Reserve, Fujian Zhangjiangkou National Mangrove Nature Reserve, Guangxi Beilun Estuary National Nature Reserve, Dongzhaigang, Huidong Harbor Sea Turtle National Nature Reserve, Guangdong Haifeng Wetlands, Guangdong Nanpeng Archipelago Wetlands, Mai Po Marshes and Inner Deep Bay, Shandong Yellow River Delta Wetland, Shanghai Yangtze Estuarine Wetland Nature Reserve for Chinese Sturgeon, Shankou Mangrove Nature Reserve, Shuangtai Estuary, Yancheng National Nature Reserve, and Zhanjiang Mangrove National Nature Reserve. All of them except Dongzhaigang have a maximum inundation extent of fewer than three times their minimum extent. However, Dongzhaigang exceptionally has its maximum inundation extent significantly greater than three times (~ 33.51 times) its minimum inundation extent (Fig. 3).
- Reservoirs/barrages are wetlands designed to maintain the desired water level with the help of a human-controllable outlet (dam/weir). However, changes in the inflow over time created variations in their inundation extent. Twenty-three wetlands come under this category, namely Xianghai, Hubei Wang Lake, Hubei Chen Lake Wetland Nature Reserve, Dong Dongting Hu, San Jiang National Nature Reserve, Dashanbao, Hubei Honghu Wetlands, Anhui Shengjin Lake National Nature Reserve, Guangdong Haifeng Wetlands, Hangzhou Xixi Wetlands, Hubei Dajiu Lake Wetland, Jilin Momoge National Nature Reserve, Mai Po Marshes and Inner Deep Bay, Shandong Yellow River Delta Wetland, Nan Dongting Wetland, and Waterfowl Nature Reserve, Shuangtai Estuary, Xingkai Lake National Nature Reserve, Xi Dongting Lake Nature Reserve, and Yancheng National Nature Reserve. Among these, all wetlands except Hubei Dajiu Lake Wetland showed variation such that their maximum inundation extent was less than four times their minimum extent. However, Hubei Dajiu Lake Wetland exceptionally has a maximum inundation extent exceeding (~ 107.8 times) its minimum inundation extent (Fig. 3).
- River stretches are wetlands that typically form part of a flowing river, their water level changes with the flow changes in the river. Henan Minquan Yellow River Gudao Wetlands, Fujian Zhangjiangkou National Mangrove Nature Reserve, Gansu Yanchiwan Wetlands, Guangxi Beilun Estuary National Nature Reserve, Yancheng National Nature Reserve, Shandong Jining Nansi Lake, Zhangye Heihe Wetland National Nature Reserve, and San Jiang National Nature Reserve were in this category. Their maximum inundation extent is 1 to 4 times their minimum inundation extent (Fig. 3).
- Natural wetlands: All the wetlands, excluding those mentioned above, lay in natural wetlands. These wetlands were marshes, swamps, lagoons, or shallow-water lakes. They function naturally and offer no control over the water content. No specific pattern was found between maximum and minimum inundation extent, but most sites have their maximum inundation value less than six times their minimum inundation value. The maximum inundation value of some sites is greater than ten times their minimum inundation value, such as Dongfanghong Wetland National Nature Reserve, Dongzhaigang, Eerduosi National Nature Reserve, Gansu Gahai Wetlands Nature Reserve, and Sichuan Ruoergai Wetland National Nature Reserve. The Guangdong Nanpeng Archipelago Wetlands (which is also a coastal wetland) displayed minimum variation with its maximum inundation extent ~ 1.01 times the minimum inundation extent, and Sichuan Changshagongma Wetlands which is also a high-altitude wetland displayed with its maximum inundation extent ~ 204.32 times the minimum inundation extent (Fig. 3).

### Trend analysis

**Figure 4:**
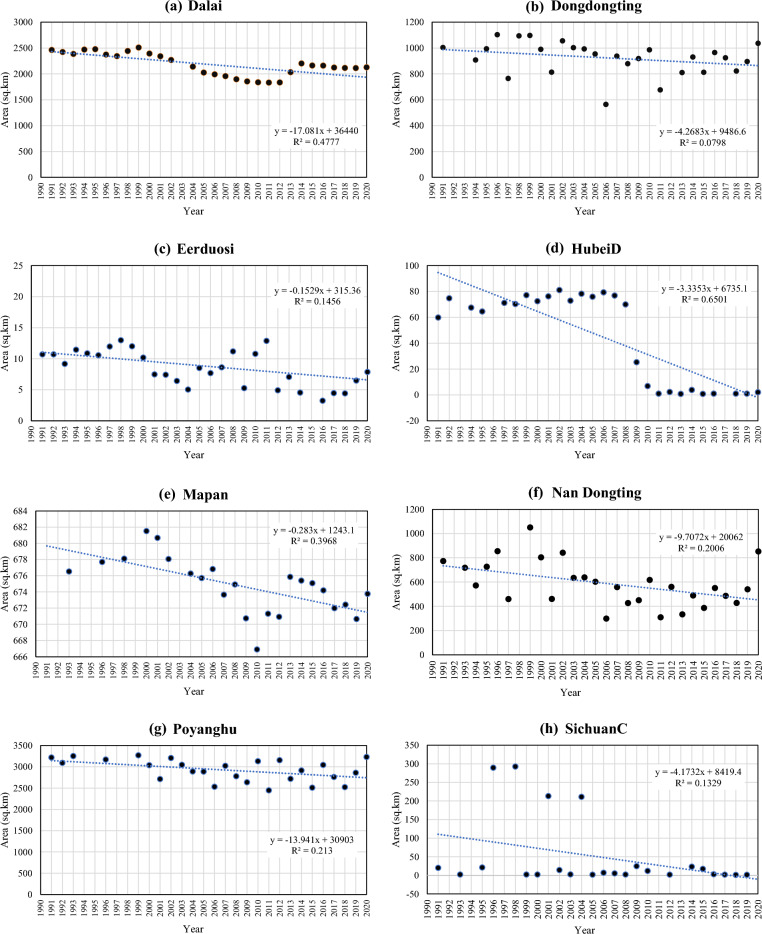
Change in inundation area for significantly decreasing Ramsar sites. The figure represents the area of each wetland for each year from 1990 to 2020. This figure is created using Microsoft Excel 365, Version 2308 (https://www.microsoft.com/en-in/microsoft-365/excel).

The Mann–Kendall (MK) test was carried out under the presumption that a significant trend is the one with a *p* value less than 0.05 (also represented by an absolute *Z*_*c*_ score greater than 1.96)^42, 43^. Since each site had a varied number of maps, the analysis was done using the information that was available, and the patterns were extrapolated to cover the entire 30-year period to allow for a consistent comparison of all the locations. Observed trends from the trend analysis suggest that 35 sites (Fig. 3) follow an increasing trend with a positive MK test statistical value (*Z*_*c*_). There are 14 out of these 35 sites (Dafeng National Nature Reserve, Eling Lake, Guangdong Haifeng Wetlands, Heilongjiang Hadong Yanjiang Wetlands, Heilongjiang Youhao Wetlands, Heilongjiang Zhenbaodao Wetland National Nature Reserve, Henan Minquan Yellow River Gudao Wetlands, Lashihai Wetland, Niaodao, Shandong Yellow River Delta Wetland, Tibet Trari Nam Co Wetlands, Xingkai Lake National Nature Reserve, Zhaling Lake, and Zhanjiang Mangrove National Nature Reserve) have a significantly increasing trend, with MK test statistical value (*Z*_*c*_) greater than + 1.96. Twenty-one out of a total of 64 sites were found to have decreasing trend with a negative MK test statistical value (*Z*_*c*_). Of these, eight sites (Dalai Lake National Nature Reserve, Dong Dongting Hu, Eerduosi National Nature Reserve, Hubei Dajiu Lake Wetland, Mapangyong Cuo, Nan Dongting Wetland, and Waterfowl Nature Reserve, Poyanghu, and Sichuan Changshagongma Wetlands) were found to have a significantly decreasing trend with MK test statistical value (Z_c_) less than − 1.96 (Fig. 4).

While the MK test is a useful tool, there are some limitations and concerns associated are: (i) the MK test does not account for seasonal patterns or repeated variations in the data. (ii) the MK test assumes that datasets are collected at equal time intervals. If the data does not meet this assumption, then data interpolation or resampling need to be performed. (iii) the trend analysis was conducted using the data available at each site. Variations in data availability can affect the accuracy of trend detection, as sites with limited data may have less robust trend assessments. (iv) for small sample sizes, the capability of the MK test to find trends may be restricted. (v) The MK test assumes that datasets are independent of each other. If there is autocorrelation (correlation between datasets at different time lags), it can lead to incorrect conclusions about the presence or absence of trends. Therefore, we conducted autocorrelation checks before employing the MK test.

**Figure 5:**
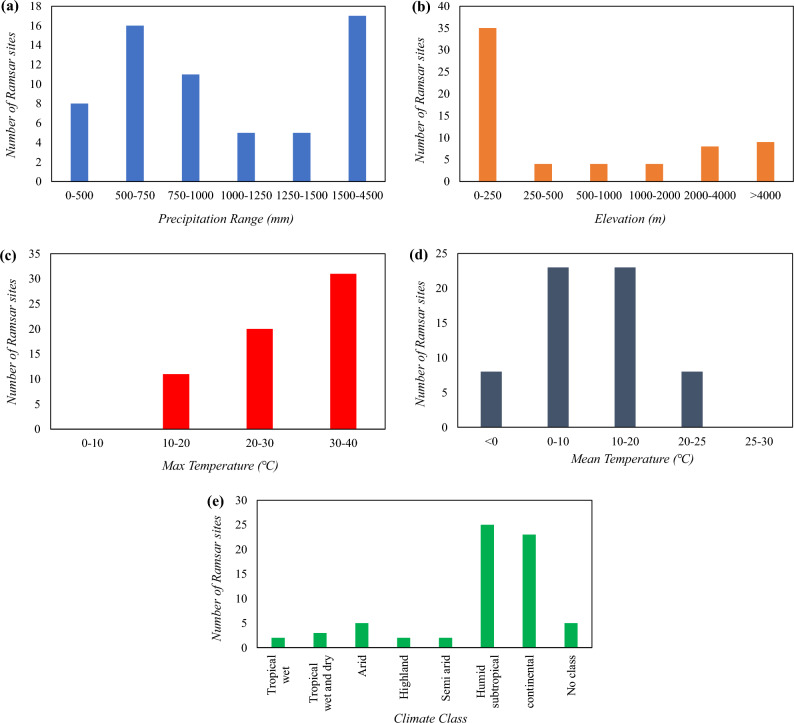
Bar chart showing the number of Ramsar sites in different ranges of (**a**) precipitation (mm), (**b**) elevation (m), (**c**) maximum temperature, (**d**) mean temperature, and (**e**) climate class. This figure is created using Microsoft Excel 365, Version 2308 (https://www.microsoft.com/en-in/microsoft-365/excel).

The patterns of trends were shown to be correlated with several variables, including mean annual precipitation, mean temperature, average annual-maximum temperature, elevation, and climatic class. Most of the sites with increasing or significantly increasing trends have elevation values less than 4000 m, except Eling Lake, Tibet Trari Nam Co Wetlands, Zhaling Lake, Maidika, and Tibet Selincuo Wetlands. Similarly, most of the sites with decreasing or significantly decreasing trends have elevation values less than 4000 m except Bitahai Wetland, Gansu Yellow River Shouqu Wetlands, Mapangyong Cuo, and Sichuan Changshagongma Wetlands (Fig. 5b).

An analogy was also found with respect to the precipitation that the wetlands received. Most of the sites with increasing or significantly increasing trends have average precipitation values greater than 500 mm, except Gansu Yanchiwan Wetlands**,** Jilin Momoge National Nature Reserve, and Tibet Trari Nam Co Wetlands. Most of the sites with decreasing trends have average precipitation values less than 1500 mm except Fujian Zhangjiangkou National Mangrove Nature Reserve, Guangxi Beilun Estuary National Nature Reserve, Huidong Harbor Sea Turtle National Nature Reserve, Jiangxi Poyang Lake Nanji Wetlands, Shankou Mangrove Nature Reserve (Fig. 5a).

It can also be inferred that most of the sites with significantly increasing trends have maximum temperature values less than 35 °C except Anhui Shengjin Lake National Nature Reserve, Hangzhou Xixi Wetlands, Hubei Wang Lake. On the other hand, most of the sites with significantly increasing trends have maximum temperature values less than 30 °C except Shandong Yellow River Delta Wetland, Henan Minquan Yellow River Gudao Wetlands, Henan Minquan Yellow River Gudao Wetlands, Dafeng National Nature Reserve. Most of the sites with decreasing or significantly decreasing trends have maximum temperature values greater than 20 °C except Bitahai Wetland, Dashanbao, Gansu Gahai Wetlands Nature Reserve, Mapangyong Cuo, Sichuan Changshagongma Wetlands (Fig. 5c).

The analogy was also found with respect to the mean Temperature of the wetlands. Most of the sites with increasing or significantly increasing trends have mean temperature values less than 15 °C except Anhui Shengjin Lake National Nature Reserve, Chongming Dongtan Nature Reserve, Hangzhou Xixi Wetlands, Hubei Chen Lake Wetland Nature Reserve, Hubei Wang Lake, Mai Po Marshes, Dafeng National Nature Reserve, Henan Minquan Yellow River Gudao Wetlands, Guangdong Haifeng Wetlands, and Inner Deep Bay. Among the sites showing increasing trends, Eling Lake, Gansu Yanchiwan Wetlands, Inner Mongolia Grand Khingan Hanma Wetlands, Maidika, Tibet Selincuo Wetlands, Zhaling Lake have negative mean temperature values. All the sites that show a significantly decreasing trend or decreasing trend, except Mapangyong Cuo, Heilongjiang Nanweng River National Nature Reserve, have their mean temperature values positive (Fig. 5d).

Ideally, a site should be decreasing if, at that site, the precipitation trend was found to be decreasing (i.e., decrease in the amount of water intake), and both mean Temperature and average annual-maximum Temperature were found to increasing (i.e., increase in the amount of water loss due to evapotranspiration and other surface water losses) and vice versa. Such an ideal relationship among the overall trend of the site, precipitation trend, maximum temperature trend, and mean temperature trend was found in the case of Dongfanghong Wetland National Nature Reserve, San Jiang National Nature Reserve, Shankou Mangrove Nature Reserve, Bitahai Wetland, Dalai Lake National Nature Reserve, Dong Dongting Hu, Dongzhaigang, Fujian Zhangjiangkou National Mangrove Nature Reserve, Guangxi Beilun Estuary National Nature Reserve, Heilongjiang Nanweng River National Nature Reserve, Hubei Dajiu Lake Wetland, Hubei Honghu Wetlands, Huidong Harbor Sea Turtle National Nature Reserve, Jiangxi Poyang Lake Nanji Wetlands, Nan Dongting Wetland, Waterfowl Nature Reserve, Poyanghu, Shanghai Yangtze Estuarine Wetland Nature Reserve for Chinese Sturgeon, Tianjin Beidagang Wetlands, Xi Dongting Lake Nature Reserve, Zhalong, and Xianghai (Fig. 5c).

### Accuracy assessment

**Figure 6:**
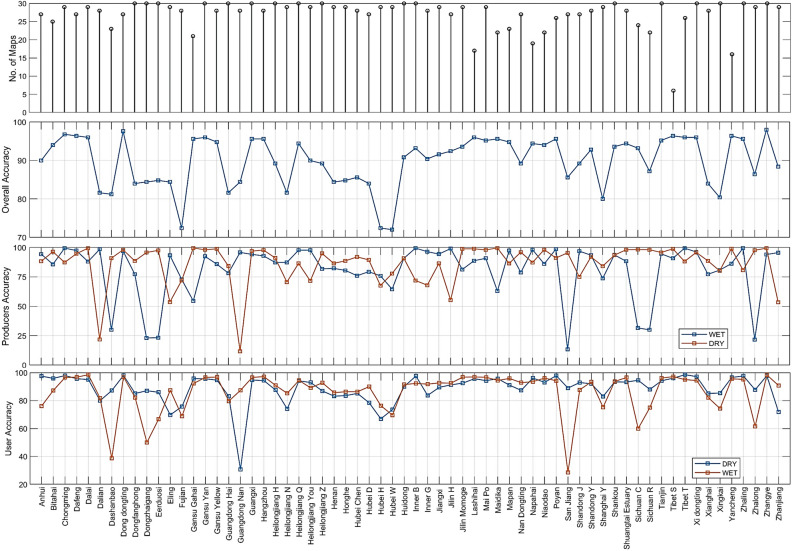
Graph showing the number of composites, overall accuracy, user accuracy, and producer accuracy for all the sites in the study. This figure is created using MATLAB R2022a (https://www.mathworks.com/products/new_products/release2022a.html).

The accuracy assessment allowed for the thresholding method to be relied upon as an effective way to produce inundation maps. Due to several circumstances, including the nature of the site, the digitizing areas, and the difference in the spectral values of wet and dry areas, the accuracy of each site turned out to be varied. The Accuracy ranged from a minimum Overall Accuracy of 72.0% at Hubei Wang Lake to a maximum of 98.0% at Zhangye Heihe Wetland National Nature Reserve. The average Overall Accuracy of the sites was found to be 90.0%, with the average dry and wet Producer's Accuracies of 86.4 and 81.3%, respectively and the dry and wet User's Accuracy of 88.7 and 86.4% (Fig. 6).

The degree of accuracy was determined by contrasting a map made from a 4-month composite with a single image from a specific day (even though it was taken from the same period), which might not accurately depict the area that was entirely inundated. It either represented less extent due to no rainfall in the near time or more due to heavy rainfall that might have happened that day, thus creating a point of error. Secondly, digitizing wet and dry areas required a human process, which was simple in wetlands with open water and parts that were alternately permanently wet and dry. However, places in those wetlands with a lot of variation in the inundated and where every part had been inundated at some point in the series made digitization difficult since it confused the classifier. The disparity between the median wet and dry values was also lessened in lakes where the water was surrounded by greenery, and the dry area was also covered with vegetation. The shadows altered the pixel values, leading to an inaccurate estimate of the median wet and dry values and a classification error. Mixed pixels may also be a source of inaccuracy. Digitizing wet and dry areas was difficult due to the marshy environment because it was difficult to make judgments from the Landsat imageries, which reduced accuracy. Due to a number of factors, including some of those stated above, the average Overall Accuracy of this study was lower than the 95.9% discovered by Inman and Lyons^27^.

## Conclusion

The effect of climatic change and commercial changes in the behavior of Ramsar wetlands has been given importance throughout the Ramsar report. This study focused on analyzing all 64 Ramsar sites in China based on their inundation pattern over the last three decades using pre-processed Landsat imageries for a period of 30 years (1991–2020). The technique of SWIR Thresholding was applied on these composites to generate inundation maps. The analysis was carried out on each Ramsar site individually to account for its typical behavior owing to regional climatic and geographical conditions. From the analysis, 37 sites showed an increasing trend, and 27 sites showed a decreasing trend. 14 out of 37 sites were significantly increasing and 8 out of 27 showed significantly decreasing behavior. The accuracy ranged from a minimum Overall Accuracy of 72.0% at Hubei Wang Lake to a maximum of 98.0% at Zhangye Heihe Wetland National Nature Reserve (Fig. 2). The average Overall Accuracy of the sites was found to be 90.0%, the average dry and wet Producer's accuracies of 86.4 and 81.3%, respectively and the dry and wet User’s Accuracy of 88.7 and 86.4%. This study helps to understand, through circumstances, the importance of wetlands and their wise management.

The limitation of study comprises various factors, including: the composites in the 1991–2004 time-period showed the most flaws, with some composites completely missing the B7 (SWIR) band due to poor Landsat 5 imageries. The coastal region adds to the complexity by forming clouds on a regular basis, making cloud masking difficult to process and resulting in regions of transparent (masked) pixels. Further, the degree of accuracy was determined by contrasting a map made from a 4-month composite with a single image from a specific day (even though it was taken from the same period), which might not accurately depict the area that was entirely inundated. The shadows altered the pixel values, leading to an inaccurate estimate of the median wet and dry values and a classification error. The mixed pixels may also be a source of inaccuracy.

For further study, the main focus would be on significantly decreasing sites, i.e., Dalai Lake National Nature Reserve, Dong Dongting Hu, Eerduosi National Nature Reserve, Hubei Dajiu Lake Wetland, Mapangyong Cuo, Nan Dongting Wetland and Waterfowl Nature Reserve, Poyanghu, and Sichuan Changshagongma Wetlands as they were found to be at the highest risk of extinction. For the conservation of these 8 sites, some machine learning models can be used to predict the future inundation of the sites and do the future analysis to find out possible reasons for the decrease in inundation area of the sites so that suitable measures can be taken to conserve the wetlands under threat. Future work could expand the management and conservation of wetlands by: (1) incorporating different satellite products at higher resolution; (2) using microwave and recently available Landsat-9 data where satellite temporal coverage is inadequate or cloud-covered; (3) mapping the spatiotemporal component of hazard with hotspots of climate impacts and risks; (4) establishing the science evidence base through recognized modelling and participatory risk assessment where modelling is not achievable; and (5) implementing actions that can lessen the vulnerability of wetlands to changing climate along with their management recommendations based on the risk assessment.

## Methods

**Figure 7:**
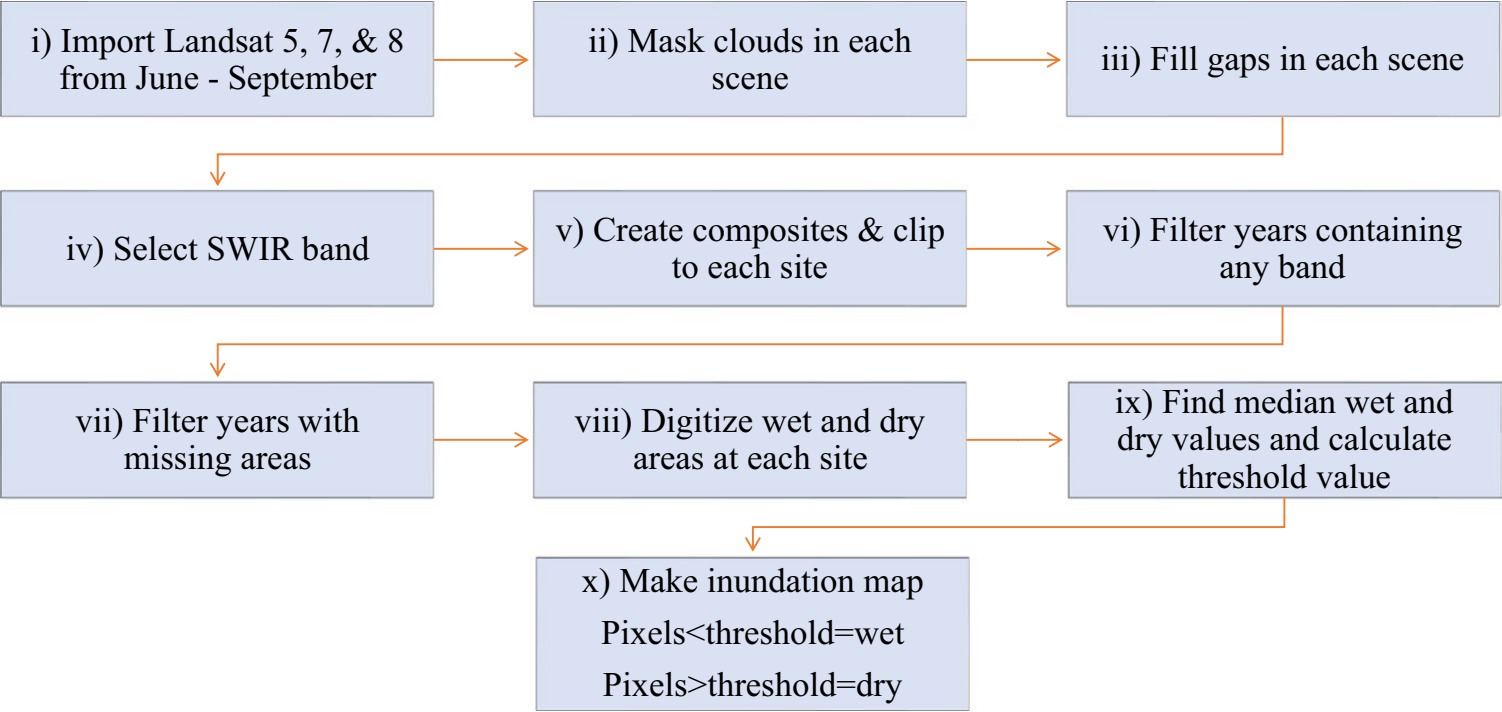
Schematic flowchart depicting the process of generating inundation maps using GEE. This figure is created using Microsoft Excel 365, Version 2308 (https://www.microsoft.com/en-in/microsoft-365/excel).

Numerous classification methods, including unsupervised, supervised band thresholding, band ratios, indices, various regression trees, and combinations of these methods, have been proposed in the past^44–46^. Out of these methods, band thresholding has distinguished itself as an effective and accurate method. Using MODIS imagery, Murray-Hudson et al. proposed a technique for thresholding the Short-Wave Infrared (SWIR) band and generated highly accurate findings^46^. The SWIR band is susceptible to moisture content on the Earth's surface. It can accurately differentiate inundated areas covered with dense vegetation from dryland^46^. This method is based on a simple formula for thresholding the SWIR band. Thus, its simplicity makes it time efficient, computationally straightforward, and readily applicable using concise algorithms; therefore, getting an edge over other complex methods of vegetation is another advantage of this method. The above-mentioned advantages of the band thresholding method are incorporated into the current study. This innovative study tries to assess all of China’s Ramsar sites simultaneously and compare their various trends and features in order to draw some conclusions. The schematic flowchart of the process of generating inundation maps using GEE is shown in Fig. 7.

### Cloud masking

Clouds and shadows can be seen in the Landsat sceneries; these elements must be hidden to produce correct composites and improve classification accuracy^47^. The pixels on the Landsat cloud mask band categorized as clouds or cloud shadows, were concealed for each scene^48–50^. The median value for the pixel from a year before or after the scenes' date was used to fill in these pixels as part of a gap-filling procedure^51, 52^.

### Landsat composites

The SWIR band (B7) is chosen for each scene, and a gap-filling method was then applied to cloud-masked images. All the scenes available from June to September of each year are used to build the composites^27, 32^. This period corresponds to the growing season in many regions when wetlands experience peak vegetation growth and dynamic changes. Monitoring wetland condition during this period provides valuable insights into vegetation health, water availability, and the overall functionality of wetland ecosystems. Most of the wetlands in China endure yearly floods that coincide with the southwest monsoon, and they are most heavily inundated from June to September^53^. The value of the relevant pixel in the composites to be formed is determined by evaluating the median of the corresponding pixel values from all the scenes of that year for each pixel in the study area.

### Filtering bad composites

The SWIR (B7) band wasn’t present in all the composites that were produced. By deleting those composites from the image collection, they were manually screened. The majority of cloud masking is handled by the algorithm. But in practically every coastal site, there were some regions where the pixels were often categorized as clouds. The masking method turned those pixels transparent in these circumstances. A filtering method was used to filter these pixels. This resulted in a set of composites devoid of masking whatsoever^54^. From site to site, a different number of final flawless composites were produced^55^. For example, an average of 27 composites were obtained for each site, with a minimum of 6 composites on Tibet Selincuo wetland and a maximum of 30 composites on Dongfanghong Wetland National Nature Reserve, Donzhaigang, Eerduosi National Nature Reserve, Gansu Yanchiwan Wetlands, Guangdong Haifeng Wetlands, Guangxi Beilun Estuary National Nature Reserve, Heilongjiang Hadong Yanjiang Wetlands, Heilongjiang Qixing River National Nature Reserve, Heilongjiang Zhenbaodao Wetland National Nature Reserve, Huidong Harbor Sea Turtle National Nature Reserve, Inner Mongolia Bila River Wetlands, Shankou Mangrove Nature Reserve, Tianjin Beidagang Wetlands, Xi Dongting Lake Nature Reserve, Xingkai Lake National Nature Reserve, Zhaling Lake, and Zhangye Heihe Wetland National Nature Reserve. Out of 64 wetlands, 60 have more than 20 composites.

### Creating inundation maps from the composites

By thresholding the SWIR band's pixel values from composite images, inundation maps are generated. First, we manually evaluated and digitalized each site's permanent wet (such as the lake's central region and permanent channels) and dry portions (like barren land or hill region near the wetland). To account for the dynamic seasonal and annual nature of the inundation patterns in the Wetlands, a composite specific $SWI{R}_{threshold}$ value is calculated using Eq. (1). Using these digitized areas, the median SWIR values for wet ($SWI{R}_{wet}$) and dry ($SWI{R}_{dry}$) inundated areas were calculated for each composite.

1 $$SWIR_{threshold} = SWIR_{wet} + 0.3 \left( {SWIR_{dry} - SWIR_{wet} } \right)$$

The classifier evaluates each pixel’s SWIR value against its $SWI{R}_{threshold}$ for each composite. Inundated pixels are those with SWIR values below $SWI{R}_{threshold}$, and dry pixels are those with SWIR values above $SWI{R}_{threshold}$. To construct an inundation map, each pixel with a certain SWIR value is categorized and translated into one of the two values, namely 0 for dry pixels and 1 for inundated pixels.

### Image-based accuracy assessment

To determine the thresholding method's applicability, particularly for China, remote sensing applications, validation was required. The simplest method might be to use Google Earth Pro's (GEP’s) historical imagery to compare inundation maps with GEP. Based on the historical imageries that are now available in GEP, a random set of five years has been chosen for each Ramsar site. The random points function in GEE was used to create a set of 50 random points for each of the five years^56^. Using GEE's Sample Region function, the pixel values at each location were extracted from the inundation maps and exported as a CSV file with just two values: 1 for inundated pixels and 0 for dry pixels. After importing the KML file into GEP, each location was evaluated visually interpretation and categorized as either dry (i.e., 0) or inundated (i.e., 1). Due to the lack of in-situ data, we had to use this as our reference dataset. This procedure was carried out at each random point in the five-year period for each site for each year. Thus, for each location, a collection of 250 random points was obtained, together with reference data from the imagery and pixel values taken from the maps. This process confirmed 16,000 points (250 × 64 = 16,000). Each site’s unique error matrix was produced. Several different types of accuracy were calculated, including Overall Accuracy (defined as the sum of the diagonal elements in the error matrix that were correctly classified and divided by the total sampled points), Producers Accuracy (defined as the diagonal entry in the error matrix for each column divided by its respective column total), and User Accuracy (defined as the diagonal entry in the error matrix for each row divided by its respective row total).

### Mann–Kendall (MK) test

The Mann–Kendall (MK) methodology is a statistical test used to find trends in time series data. The MK test was run on each site separately to look for trends in the variation of inundation extent. This test was initially developed by Mann et al. in^57^ and was further investigated by Kendall et al. in^58^ and by Hirsch et al. in^59^. Blain et al. provided an assessment^42^. The MK test generates test statistics, which follows a known probability distribution, allowing for the calculation of *p* values. The MK test was carried out under the presumption that a significant trend is the one with a *p* value less than 0.05 (also represented by an absolute *Z*_*c*_ score greater than 1.96)^42^. The *p* value is used to assess the significance of any observed trend and if less than a chosen significance level (typically 0.05) indicates a statistically significant trend, suggesting that the observed pattern is unlikely to be due to random chance^42, 43^.

### Supplementary Information

Supplementary Information.

## Data Availability

To get the entire time series dataset from 1991 to 2020, we employed all three Landsat sensor imageries (Landsat 5 TM, Landsat 7 ETM+, and Landsat 8 OLI). Landsat 5 (availability: 1984 to 2012) and Landsat 7 (availability: 1999 to present) images contain four Visible and Near-Infrared (VNIR) bands and two short-wave infrared (SWIR or B7) bands processed to orthorectified surface reflectance, and one thermal infrared (TIR) band processed to orthorectified brightness temperature, while Landsat 8 (2013 to present) contains 11 bands. The SWIR bands in all the Landsat scenes have a 30 m/pixel resolution. The GEE has all the above datasets available in its data catalog and ready for usage (https://developers.google.com/earth-engine/datasets/catalog/LANDSAT_LT05_C01_T1_SR; https://developers.google.com/earth-engine/datasets/catalog/LANDSAT_LE07_C01_T1_SR; https://developers.google.com/earth-engine/datasets/catalog/LANDSAT_LC08_C01_T1_SR). For the analysis, most of the shapefiles of the wetlands were obtained from the official Ramsar website (https://rsis.ramsar.org/). However, the shapefiles of Bitahai Wetland, Eerduosi National Nature Reserve, Jilin Momoge National Nature Reserve, Niaodao, Sichuan Ruoergai Wetland National Nature Reserve, Xi Dongting Lake Nature Reserve, and Zhanjiang Mangrove National Nature Reserve were manually created in QGIS using the coordinate information available on the official website at a scale of 10,000. Climate data such as average precipitation, maximum temperature, mean temperature, and average precipitation for each wetland, has been extracted from ECMWF reanalysis data (ERA5 monthly)^60^ using GEE (https://developers.google.com/earth-engine/datasets/catalog/ECMWF_ERA5_MONTHLY).

## Code availability

The GEE allows the inundation mapping algorithm to be applied on Landsat images. Codes for inundation mapping and other parts of the analysis will be made available upon request to the corresponding author.

## References

[1] Davidson NC (2014). How much wetland has the world lost? Long-term and recent trends in global wetland area. Mar. Freshw. Res..

[2] Bar-On YM, Phillips R, Milo R (2018). The biomass distribution on Earth. Proc. Natl. Acad. Sci..

[3] Scheyer TM (2013). Crocodylian diversity peak and extinction in the late Cenozoic of the northern Neotropics. Nat. Commun..

[4] Kulshreshtha S, Sharma BK, Sharma S (2013). The Ramsar sites of Rajasthan: Ecology and conservation of Sambhar Salt Lake, Jaipur and Keoladeo National Park, Bharatpur. Faunal Heritage of Rajasthan, India.

[5] Rathod DM, Parasharya BM (2018). Odonate diversity of Nalsarovar Bird Sanctuary-a Ramsar site in Gujarat, India. J. Threat. Taxa.

[6] Mitsch WJ, Bernal B, Hernandez ME (2015). Ecosystem services of wetlands. Int. J. Biodivers. Sci. Ecosyst. Serv. Manag..

[7] Fluet-Chouinard E (2023). Extensive global wetland loss over the past three centuries. Nature.

[8] Davidson NC, Finlayson CM (2018). Extent, regional distribution and changes in area of different classes of wetland. Mar. Freshw. Res..

[9] Courouble, M., Davidson, N., Dinesen, L., Fennessy, S., Galewski, T., Guelmami, A., Kumar, R., McInnes, R., Perennou, C., Rebelo, L. M., Robertson, H., Segura-Champagnon, M. S. *Global Wetland Outlook: Special Edition 2021*. (2021). https://static1.squarespace.com/static/5b256c78e17ba335ea89fe1f/t/61b8a904f3ceb458e9b5ca44/1639491853578/Ramsar+GWO_Special+Edition+2021%E2%80%93ENGLISH_WEB.pdf.

[10] Haig SM, Murphy SP, Matthews JH, Arismendi I, Safeeq M (2019). Climate-altered wetlands challenge waterbird use and migratory connectivity in arid landscapes. Sci. Rep..

[11] Xi Y, Peng S, Ciais P, Chen Y (2021). Future impacts of climate change on inland Ramsar wetlands. Nat. Clim. Change.

[12] Frazier, S. Ramsar sites overview. *Wetl. Int.* (1999).

[13] Ramsar. Scaling up wetland conservation, wise use and restoration to achieve the Sustainable Development Goals. https://www.ramsar.org/sites/default/files/documents/library/wetlands_sdgs_e.pdf (2018).

[14] Jaramillo F (2019). Priorities and Interactions of Sustainable Development Goals (SDGs) with Focus on Wetlands. Water.

[15] Mao D, Wang Z, Wang Y, Choi CY, Jia M, Jackson MV, Fuller RA (2021). Remote observations in China’s Ramsar Sites: Wetland dynamics, anthropogenic threats, and implications for sustainable development goals. J. Remote Sens..

[16] Poonia V, Jha S, Goyal MK (2021). Copula based analysis of meteorological, hydrological and agricultural drought characteristics across Indian river basins. Int. J. Climatol..

[17] Mao D (2020). National wetland mapping in China: A new product resulting from object-based and hierarchical classification of Landsat 8 OLI images. ISPRS J. Photogramm. Remote Sens..

[18] Ramsar. Annotated List of Wetlands of International Importance: China. https://www.ramsar.org/wetland/china (2022).

[19] Niu Z, Zhang H, Gong P (2011). More protection for China’s wetlands. Nature.

[20] Wang Z, Wu J, Madden M, Mao D (2012). China’s wetlands: Conservation plans and policy impacts. Ambio.

[21] Xu W (2019). Transforming protected area management in China. Trends Ecol. Evol..

[22] Gong P (2010). China’s wetland change (1990–2000) determined by remote sensing. Sci. China Earth Sci..

[23] Niu Z (2012). Mapping wetland changes in China between 1978 and 2008. Chin. Sci. Bull..

[24] Mao D (2018). Conversions between natural wetlands and farmland in China: A multiscale geospatial analysis. Sci. Total Environ..

[25] Goyal MK, Gupta AK, Jha S, Rakkasagi S, Jain V (2022). Climate change impact on precipitation extremes over Indian cities: Non-stationary analysis. Technol. Forecast. Soc. Change.

[26] Dottori F (2018). Increased human and economic losses from river flooding with anthropogenic warming. Nat. Clim. Change.

[27] Inman VL, Lyons MB (2020). Automated inundation mapping over large areas using Landsat data and Google Earth Engine. Remote Sens..

[28] Shirzaei M, Bürgmann R (2018). Global climate change and local land subsidence exacerbate inundation risk to the San Francisco Bay Area. Sci. Adv..

[29] Valenti VL, Carcelen EC, Lange K, Russo NJ, Chapman B (2020). Leveraging Google Earth Engine user interface for semiautomated wetland classification in the Great Lakes Basin at 10 m with optical and radar geospatial datasets. IEEE J. Sel. Top. Appl. Earth Obs. Remote Sens..

[30] Schumann G, Di Baldassarre G, Bates PD (2009). The utility of spaceborne radar to render flood inundation maps based on multialgorithm ensembles. IEEE Trans. Geosci. Remote Sens..

[31] Khalaf M (2020). IoT-enabled flood severity prediction via ensemble machine learning models. IEEE Access.

[32] Amani M (2021). Wetland change analysis in Alberta, Canada using four decades of Landsat imagery. IEEE J. Sel. Top. Appl. Earth Obs. Remote Sens..

[33] Zurqani HA (2020). Evaluating the integrity of forested riparian buffers over a large area using LiDAR data and Google Earth Engine. Sci. Rep..

[34] Ahmad SK, Hossain F, Eldardiry H, Pavelsky TM (2020). A fusion approach for water area classification using visible, near infrared and synthetic aperture radar for South Asian conditions. IEEE Trans. Geosci. Remote Sens..

[35] Dubey S, Gupta H, Goyal MK, Joshi N (2021). Evaluation of precipitation datasets available on Google earth engine over India. Int. J. Climatol..

[36] Goyal MK, Sharma A, Surampalli RY (2020). Remote sensing and GIS applications in sustainability. Sustainability.

[37] Gallant AL (2015). The challenges of remote monitoring of wetlands. Remote Sens..

[38] Lin Y, Cui Q, Li H, He C (2022). Assessment of importance of 64 Ramsar sites in China for waterfowl. Glob. Ecol. Conserv..

[39] Meng W (2017). Status of wetlands in China: A review of extent, degradation, issues and recommendations for improvement. Ocean Coast. Manag..

[40] Thito K, Wolski P, Murray-Hudson M (2016). Mapping inundation extent, frequency and duration in the Okavango Delta from 2001 to 2012. Afr. J. Aquat. Sci..

[41] Gupta V, Rakkasagi S, Rajpoot S, El Imanni HS, Singh S (2023). Spatiotemporal analysis of Imja Lake to estimate the downstream flood hazard using the SHIVEK approach. Acta Geophys..

[42] Blain GC (2013). The Mann-Kendall test: The need to consider the interaction between serial correlation and trend. Acta Sci. Agron..

[43] Pal I, Al-Tabbaa A (2009). Trends in seasonal precipitation extremes–An indicator of ‘climate change’ in Kerala, India. J. Hydrol..

[44] Gumbricht T, Wolski P, Frost P, McCarthy T (2004). Forecasting the spatial extent of the annual flood in the Okavango delta, Botswana. J. Hydrol..

[45] Milzow C, Kgotlhang L, Kinzelbach W, Meier P, Bauer-Gottwein P (2009). The role of remote sensing in hydrological modelling of the Okavango Delta, Botswana. J. Environ. Manag..

[46] Murray-Hudson M (2015). Remote Sensing-derived hydroperiod as a predictor of floodplain vegetation composition. Wetl. Ecol. Manag..

[47] Subramoniam SR, Ravindranath S, Rakkasagi S, Ram H (2022). Water resource management studies at micro level using geospatial technologies. Geospatial Technologies for Resources Planning and Management.

[48] Dubey S, Goyal MK (2020). Glacial lake outburst flood hazard, downstream impact, and risk over the Indian Himalayas. Water Resour. Res..

[49] Goswami UP, Goyal MK (2021). Assessment of glacial lake development and downstream flood impacts of critical glacial lake. Nat. Hazards.

[50] McCarthy JM (2003). Flooding patterns of the Okavango wetland in Botswana between 1972 and 2000. Ambio A J. Hum. Environ..

[51] Mahdianpari M (2020). Big data for a big country: The first generation of Canadian Wetland inventory map at a spatial resolution of 10-m using Sentinel-1 and Sentinel-2 data on the Google Earth Engine cloud computing platform. Can. J. Remote Sens..

[52] Mahdianpari M, Salehi B, Mohammadimanesh F, Homayouni S, Gill E (2018). The first wetland inventory map of newfoundland at a spatial resolution of 10 m using Sentinel-1 and Sentinel-2 data on the google earth engine cloud computing platform. Remote Sens..

[53] Kumar V, Jain SK, Singh Y (2010). Analysis of long-term rainfall trends in India. Hydrol. Sci. J..

[54] Bourgeau-Chavez L (2015). Development of a bi-national Great Lakes coastal wetland and land use map using three-season PALSAR and Landsat imagery. Remote Sens..

[55] Yin G, Mariethoz G, McCabe M (2016). Gap-filling of Landsat 7 imagery using the direct sampling method. Remote Sens..

[56] Gupta SK, Shukla DP (2016). Assessment of land use/land cover dynamics of Tso Moriri Lake, a Ramsar site in India. Environ. Monit. Assess..

[57] Mann HB (1945). Nonparametric tests against trend. Econometrica.

[58] Stuart A (1956). Rank correlation methods. Br. J. Stat. Psychol..

[59] Hirsch RM, Slack JR (1984). A nonparametric trend test for seasonal data with serial dependence. Water Resour. Res..

[60] Sharma A, Goyal MK (2018). District-level assessment of the ecohydrological resilience to hydroclimatic disturbances and its controlling factors in India. J. Hydrol..

