# Supplementary material for: Spatiotemporal-based automated inundation mapping of Ramsar wetlands using Google Earth Engine
Source: Sci Rep. 2023 Oct 13;13:17324. doi: 10.1038/s41598-023-43910-4 (PMC10575872; doi:10.1038/s41598-023-43910-4)
Supplement: Supplementary file 1 — Supplementary Information. [file 41598_2023_43910_MOESM1_ESM.docx]

Supplementary Materials for

**Spatiotemporal-based automated inundation mapping of Ramsar wetlands using Google Earth Engine**

Manish Kumar Goyal *et al.*

* Corresponding author – Email ID: [mkgoyal@iiti.ac.in](mailto:mkgoyal@iiti.ac.in)

**S1. Criteria for identification of wetland as a Ramsar site**

**Table S1**. The Ramsar Criteria declares that wetlands must be selected based on their individuality and international significance in terms of the biodiversity and their botanical, zoological, and hydrological features. If any of the following nine criteria apply to a wetland^1,2^, it might be declared internationally important.

| **Criteria** | **Basis of Selection** |
| --- | --- |
| 1 | It comprises a representative, uncommon, or unique example of a natural or near-natural wetland type located within the suitable biogeographic area. |
| 2 | It sustains susceptible, endangered, or severely endangered species or threatened natural communities. |
| 3 | It maintains populations of plant and/or animal species crucial for preserving the ecological diversity of a specific biogeographic region. |
| 4 | It sustains plant and/or animal species at a vital point in their life cycles or offers sanctuary under harsh conditions. |
| 5 | It consistently sustains 20,000 or more waterbirds. |
| 6 | It routinely supports 1% of the individuals in a population of one species or subspecies of waterbird. |
| 7 | It sustains many indigenous fish subspecies, species or families, life-history stages, species interactions, and/or populations that are typical of wetland benefits and/or values, and hence contributes to world biological diversity. |
| 8 | It is a major source of food for fishes, a spawning site, nursery, and/or migration corridor on which fish stocks, whether inside the wetland or outside, rely. |
| 9 | It routinely maintains 1% of the individuals in a population of one species or subspecies of wetland-dependent non-avian animal species. |

**S2. Inundation maps of wetlands in China**

The inundation maps of the remaining 49 Ramsar sites are presented in Fig. S2a−d. These figures are created using QGIS 3.30.1 (Quantum GIS; <https://download.qgis.org/downloads/>).

**
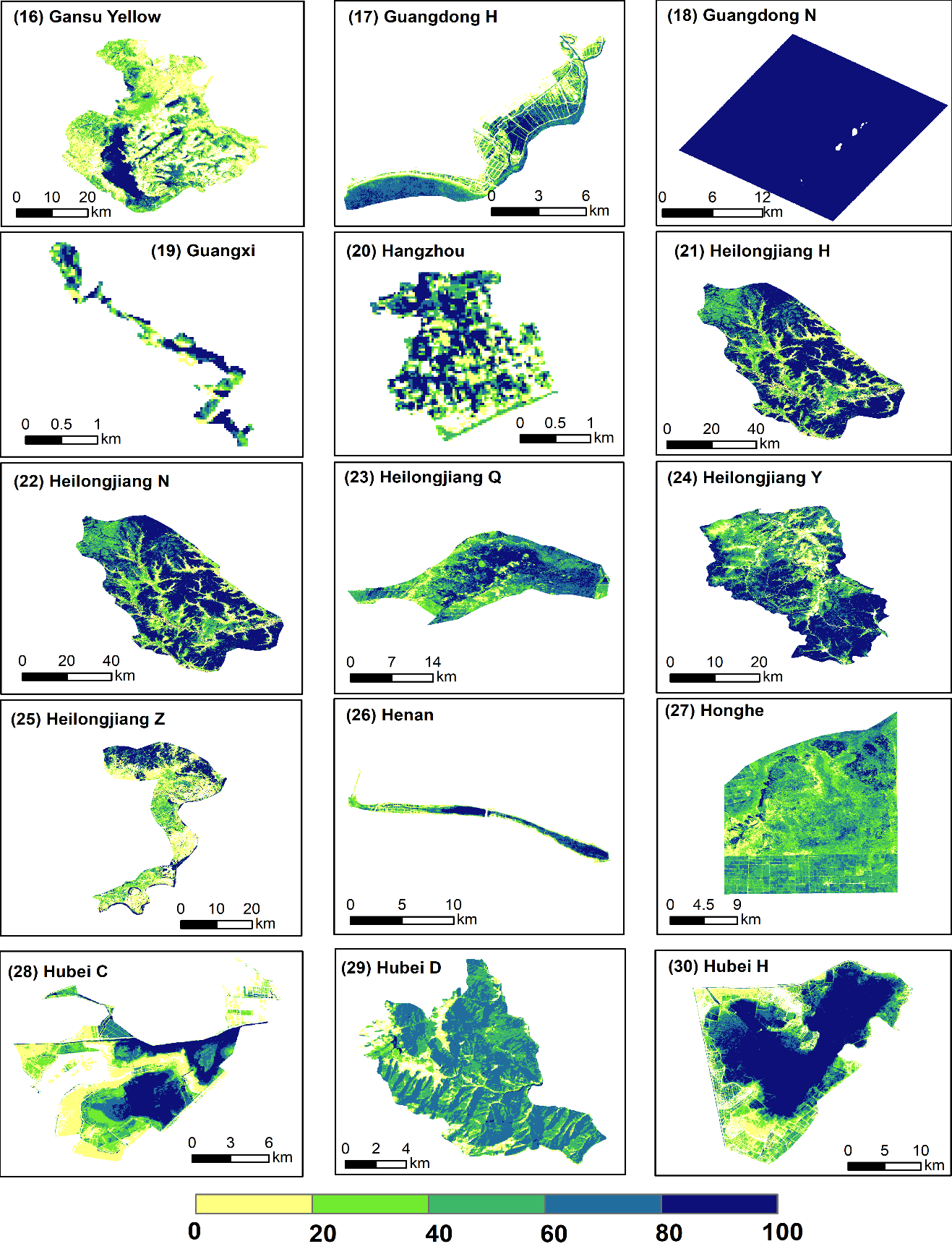
**

**Fig. S2(a)**: Inundation maps represent the next 15 out of remaining 49 Ramsar sites in China.

**
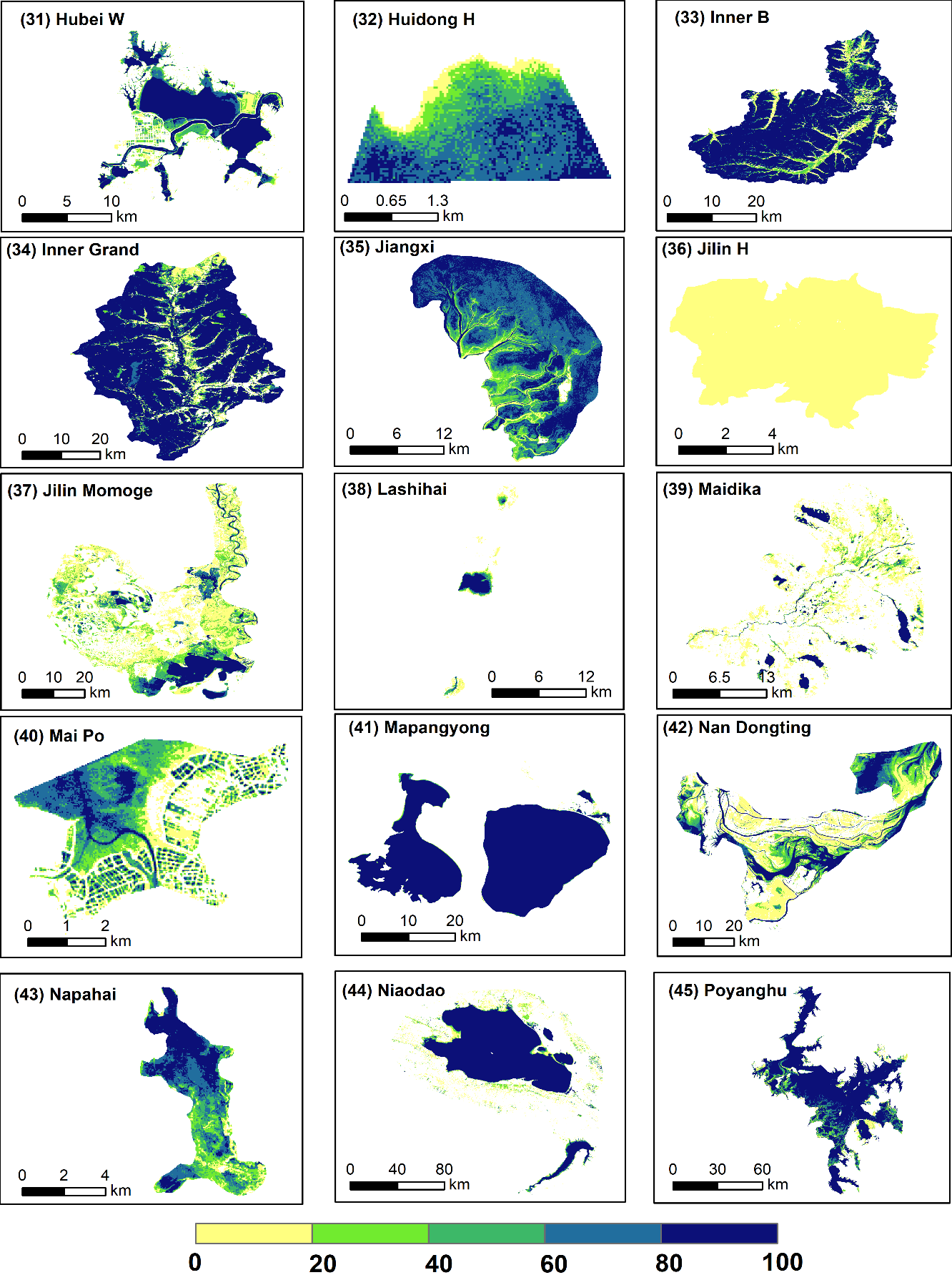
**

**Fig. S2(b)**: Inundation maps represent the next 15 out of remaining 49 Ramsar sites in China.

**
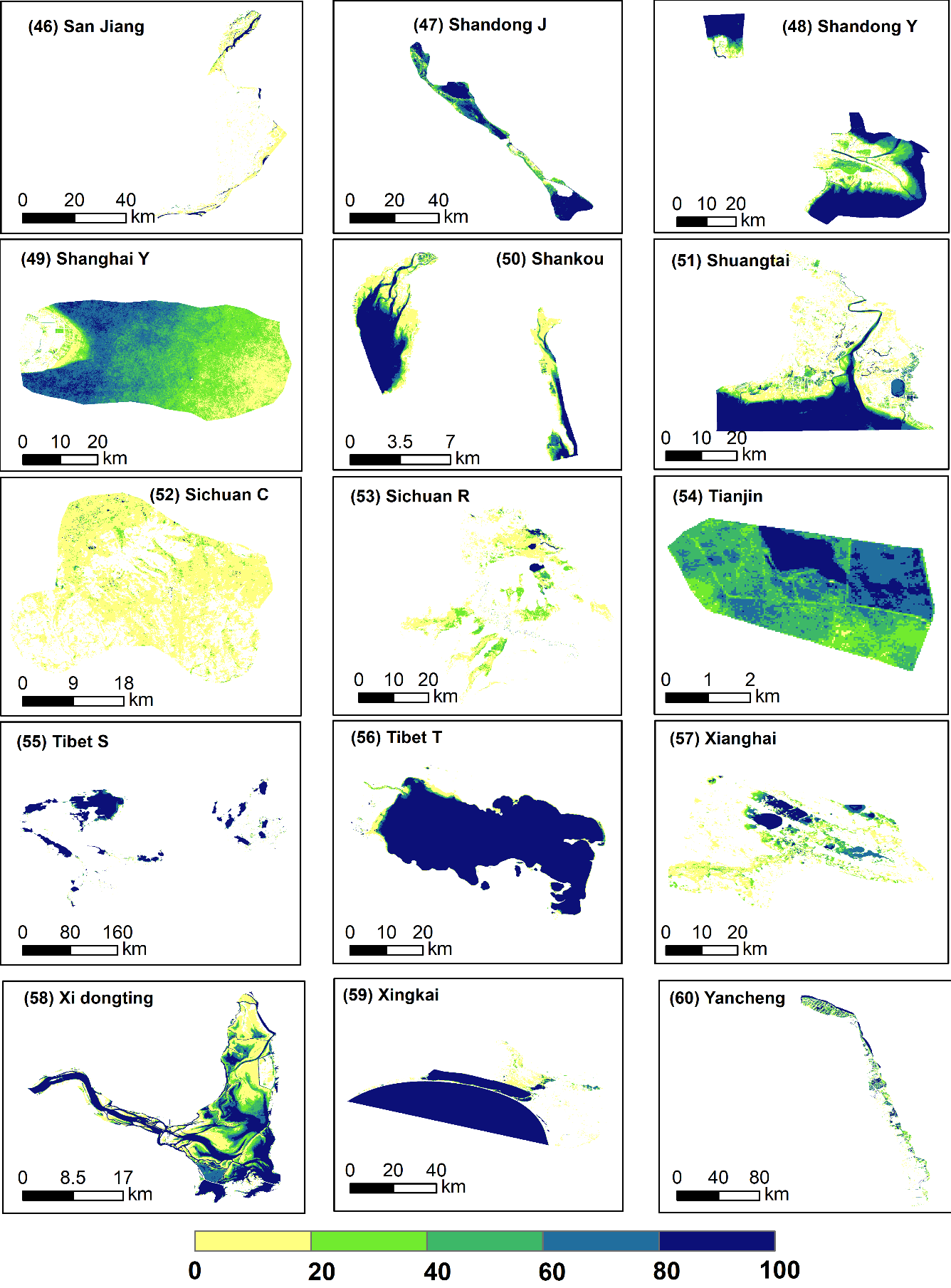
**

**Fig. S2(c)**: Inundation maps represent the next 15 out of remaining 49 Ramsar sites in China.

**
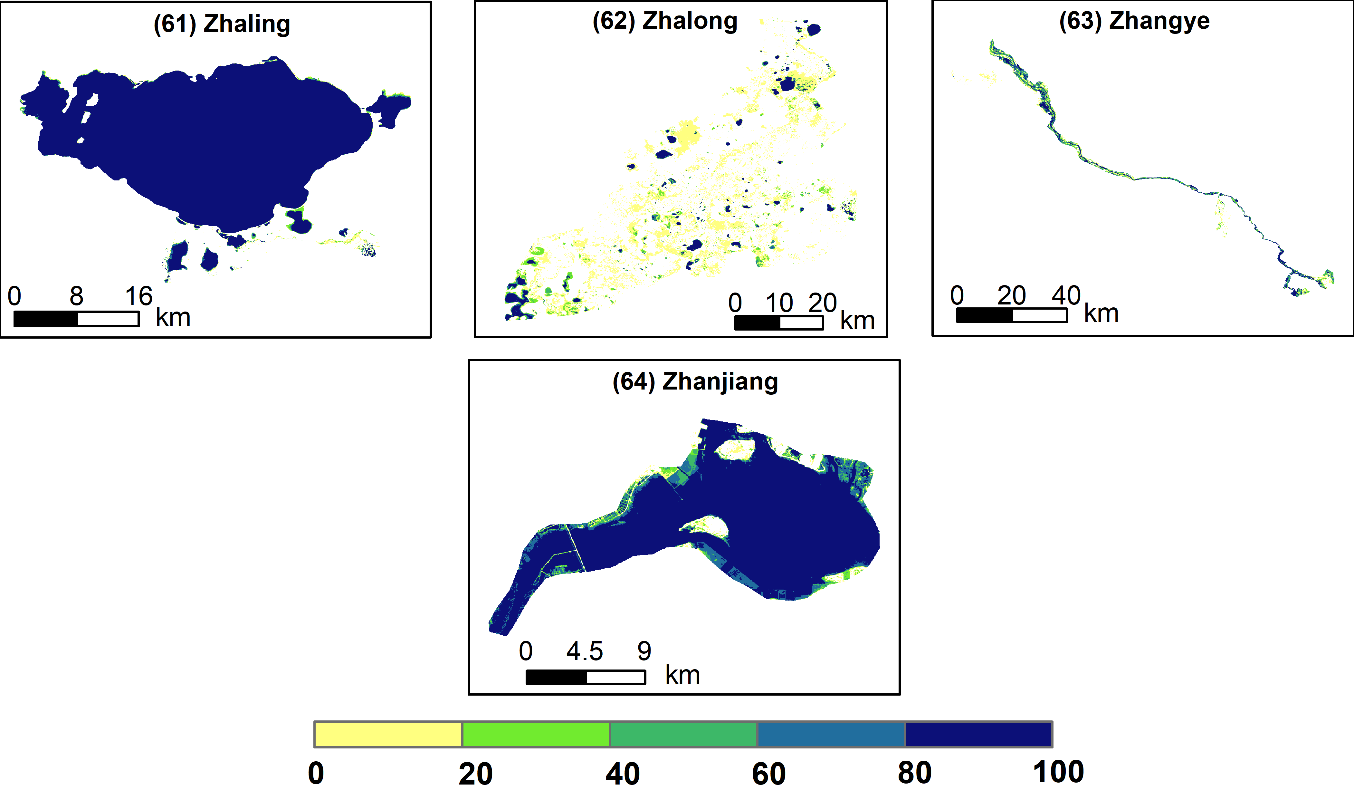
**

**Fig. S2(d)**: Inundation maps represent the last 4 Ramsar sites in China.

**S3. Details of each Ramsar site of China**

**Table S3a**. The table shows the area and criteria used to identify as a Ramsar site for each wetland in China along with their respective site names.

| **Site name** | **Area (ha)** | **Criteria** | | | | | | | | | |
| --- | --- | --- | --- | --- | --- | --- | --- | --- | --- | --- | --- |
|  |  | **1** | **2** | **3** | **4** | **5** | **6** | **7** | **8** | **9** |  |
| Xianghai | 105467 | Yes | Yes | No | No | Yes | Yes | No | No | No |  |
| Zhalong | 210000 | Yes | Yes | No | No | Yes | Yes | No | No | No |  |
| Dalian National Spotted Seal | 11700 | No | Yes | Yes | No | No | No | No | No | No |  |
| Gansu Yanchiwan Wetlands | 29876.2 | Yes | Yes | Yes | Yes | Yes | Yes | Yes | No | No |  |
| Hubei Wang Lake | 20495 | Yes | Yes | Yes | Yes | Yes | Yes | Yes | Yes | No |  |
| Hubei Chen Lake Wetland | 11579 | Yes | Yes | No | Yes | Yes | Yes | No | No | No |  |
| Dafeng National Nature Reserve | 78000 | No | Yes | Yes | No | Yes | No | No | No | Yes |  |
| Sichuan Changshagongma Wetlands | 669800 | Yes | Yes | Yes | Yes | No | Yes | Yes | No | No |  |
| Dong dongting hu | 190000 | Yes | Yes | Yes | Yes | Yes | Yes | No | No | No |  |
| Guangxi Beilun Estuary | 3000 | Yes | Yes | Yes | Yes | No | No | No | Yes | No |  |
| San Jiang National Nature Reserve | 164400 | Yes | Yes | Yes | Yes | Yes | No | Yes | Yes | No |  |
| Dongzhaigang | 5400 | Yes | No | Yes | No | No | No | No | No | No |  |
| Dashanbao | 5958 | No | Yes | Yes | Yes | No | Yes | No | No | No |  |
| Heilongjiang Youhao Wetlands | 60687 | Yes | Yes | Yes | Yes | Yes | Yes | Yes | Yes | No |  |
| Zhaling Lake | 64920 | Yes | Yes | Yes | No | No | No | Yes | Yes | No |  |
| Huidong Harbor Sea Turtle | 400 | Yes | Yes | No | Yes | No | No | No | No | No |  |
| Jilin Hani Wetlands | 3571.5 | Yes | Yes | Yes | Yes | No | No | Yes | No | No |  |
| Inner Mongolia Grand Khingan Hanma | 107348 | Yes | Yes | Yes | Yes | No | No | No | No | No |  |
| Tibet Selincuo Wetlands | 1893630 | Yes | Yes | Yes | Yes | Yes | Yes | No | No | No |  |
| Hubei Honghu Wetlands | 43450 | Yes | Yes | Yes | No | Yes | Yes | Yes | No | No |  |
| Anhui Shengjin Lake | 33340 | No | Yes | Yes | Yes | Yes | Yes | No | No | No |  |
| Bitahai Wetland | 1985 | Yes | Yes | Yes | Yes | No | No | Yes | Yes | No |  |
| Dongfanghong Wetland | 31538 | Yes | Yes | No | Yes | Yes | Yes | Yes | No | No |  |
| Eling Lake | 65907 | Yes | Yes | Yes | No | No | No | Yes | Yes | No |  |
| Dalai Lake, Inner Mongolia | 740000 | No | Yes | No | No | Yes | Yes | Yes | Yes | No |  |
| Eerduosi National Nature Reserve | 7680 | Yes | Yes | Yes | No | Yes | Yes | No | No | No |  |
| Gansu Gahai Wetlands Nature Reserve | 247431 | Yes | Yes | Yes | Yes | Yes | Yes | No | No | No |  |
| Guangdong Haifeng Wetlands | 11590.5 | Yes | Yes | No | Yes | Yes | Yes | No | No | No |  |
| Guangdong Nanpeng Archipelago | 35679 | Yes | Yes | Yes | Yes | No | No | Yes | Yes | No |  |
| Hangzhou Xixi Wetlands | 325 | Yes | Yes | No | No | No | No | Yes | No | No |  |
| Heilongjiang Nanweng River | 229523 | Yes | Yes | Yes | Yes | Yes | Yes | No | No | No |  |
| Heilongjiang Qixing River | 20000 | Yes | Yes | Yes | Yes | Yes | Yes | No | No | No |  |
| Heilongjiang Zhenbaodao Wetland | 44364 | Yes | Yes | Yes | Yes | Yes | Yes | No | No | No |  |
| Honghe National Nature Reserve | 21836 | Yes | Yes | No | Yes | Yes | Yes | No | No | No |  |
| Hubei Dajiu Lake Wetland | 9320 | Yes | Yes | Yes | No | No | No | No | No | No |  |
| Jilin Momoge National Nature Reserve | 144000 | Yes | Yes | Yes | No | Yes | Yes | Yes | No | No |  |
| Lashihai Wetland | 3560 | Yes | Yes | Yes | Yes | Yes | No | No | No | No |  |
| Mai Po Marshes and Inner Deep Bay | 1540 | No | Yes | Yes | No | Yes | Yes | No | No | No |  |
| Mapangyong Cuo | 73782 | Yes | Yes | Yes | Yes | Yes | No | Yes | Yes | No |  |
| Napahai Wetland | 2083 | Yes | Yes | Yes | Yes | Yes | Yes | No | No | No |  |
| Niaodao | 53600 | Yes | Yes | No | No | Yes | Yes | No | No | No |  |
| Poyanghu | 22400 | Yes | Yes | No | No | Yes | Yes | No | No | No |  |
| Shandong Yellow River Delta Wetland | 95950 | Yes | Yes | No | Yes | Yes | Yes | No | No | No |  |
| Shanghai Yangtze Estuarine Wetland | 3760 | Yes | Yes | No | Yes | No | No | Yes | Yes | No |  |
| Shankou Mangrove Nature Reserve | 4000 | Yes | Yes | Yes | Yes | No | No | No | No | No |  |
| Nan Dongting Wetland and Waterfowl | 168000 | No | Yes | Yes | No | Yes | Yes | No | No | No |  |
| Shuangtai Estuary | 128000 | Yes | Yes | Yes | Yes | Yes | Yes | Yes | Yes | No |  |
| Xingkai Lake National Nature Reserve | 222488 | Yes | Yes | Yes | Yes | Yes | Yes | Yes | Yes | No |  |
| Sichuan Ruoergai Wetland | 166570 | Yes | Yes | Yes | Yes | No | Yes | Yes | No | No |  |
| Xi dongting lake nature reserve | 35000 | Yes | Yes | Yes | Yes | Yes | Yes | No | No | No |  |
| Yancheng National Nature Reserve | 453000 | No | Yes | Yes | Yes | Yes | Yes | Yes | No | No |  |
| Zhangye Heihe Wetland | 41164.56 | Yes | Yes | No | Yes | Yes | Yes | No | No | No |  |
| Zhanjiang Mangrove | 20279 | Yes | Yes | Yes | No | No | No | No | No | No |  |
| Chongming Dongtan, Shanghai | 32600 | Yes | Yes | Yes | Yes | Yes | Yes | Yes | Yes | No |  |
| Fujian Zhangjiangkou Mangrove | 2358 | Yes | Yes | Yes | No | No | No | No | Yes | No |  |
| Shandong Jining Nansi Lake | 50761.56 | Yes | Yes | Yes | Yes | No | No | Yes | No | No |  |
| Tianjin Beidagang Wetlands | 1130 | No | Yes | No | No | Yes | Yes | No | No | No |  |
| Henan Minquan Yellow River Gudao | 2303.5 | No | Yes | No | Yes | Yes | Yes | No | No | No |  |
| Heilongjiang Hadong Yanjiang | 9973.62 | No | Yes | No | No | Yes | Yes | Yes | No | No |  |
| Tibet Trari Nam Co Wetlands | 142982 | Yes | Yes | No | No | Yes | Yes | No | No | No |  |
| Inner Mongolia Bila River Wetlands | 56604 | Yes | Yes | Yes | No | No | No | No | No | No |  |
| Jiangxi Poyang Lake Nanji Wetlands | 33300 | Yes | Yes | No | No | Yes | Yes | No | No | No |  |
| Maidika | 43496 | Yes | Yes | No | No | No | No | No | No | No |  |
| Gansu Yellow River Shouqu Wetlands | 132067 | Yes | Yes | Yes | Yes | No | No | No | No | No |  |

**Table S3b**. The table shows the latitude, longitude, wetland type, maximum elevation, and minimum elevation for each wetland in China.

| **Site name** | **Latitude** | **Longitude** | **Wetland type** | **Maximum elevation** | **Minimum elevation** |
| --- | --- | --- | --- | --- | --- |
| Xianghai | 45.03333 | 122.68333 | Human-made | 192 | 156 |
| Zhalong | 47.20000 | 124.20000 | Inland | 146 | 140 |
| Dalian National Spotted Seal | 39.09200 | 121.26307 | Marine or coastal | 329 | 0 |
| Gansu Yanchiwan Wetlands | 39.09149 | 95.83791 | Inland | 3300 | 3100 |
| Hubei Wang Lake | 29.84370 | 115.33346 | Inland | 440 | 7 |
| Hubei Chen Lake Wetland | 30.33353 | 113.82606 | Human-made | 21 | 18 |
| Dafeng National Nature Reserve | 33.26822 | 120.79335 | Marine or coastal | 2 | 1 |
| Sichuan Changshagongma Wetlands | 33.76004 | 97.99155 | Inland | 5249 | 3840 |
| Dong dongting hu | 29.32987 | 112.95375 | Inland | 35 | 18 |
| Guangxi Beilun Estuary | 21.59085 | 108.15691 | Marine or coastal | 2 | 1 |
| San Jiang National Nature Reserve | 47.93333 | 134.33333 | Inland | 50 | 0 |
| Dongzhaigang | 19.98333 | 110.58333 | Marine or coastal | 0 | 0 |
| Dashanbao | 27.42660 | 103.32552 | Inland | 3364 | 2210 |
| Heilongjiang Youhao Wetlands | 48.40260 | 128.36734 | Inland | 546 | 436 |
| Zhaling Lake | 34.91164 | 97.27479 | Inland | 4500 | 4200 |
| Huidong Harbor Sea Turtle | 22.55000 | 114.90000 | Marine or coastal | 25 | -10 |
| Jilin Hani Wetlands | 42.21448 | 126.51916 | Inland | 906 | 887 |
| Inner Mongolia Grand Khingan Hanma | 51.58905 | 122.63364 | Inland | 1418 | 824 |
| Tibet Selincuo Wetlands | 31.37484 | 89.59196 | Inland | 5070 | 4540 |
| Hubei Honghu Wetlands | 29.83333 | 113.31667 | Human-made | 28 | 21 |
| Anhui Shengjin Lake | 30.38069 | 117.08827 | Inland | 25 | 1 |
| Bitahai Wetland | 27.70000 | 100.01667 | Inland | 4260 | 3000 |
| Dongfanghong Wetland | 46.30963 | 133.74917 | Inland | 312 | 47 |
| Eling Lake | 34.90662 | 97.68028 | Inland | 4500 | 4200 |
| Dalai Lake, Inner Mongolia | 48.75498 | 117.47896 | Inland | 784 | 545 |
| Eerduosi National Nature Reserve | 39.80000 | 109.58333 | Inland | 1520 | 1360 |
| Gansu Gahai Wetlands Nature Reserve | 34.27778 | 102.44806 | Inland | 3757 | 3000 |
| Guangdong Haifeng Wetlands | 22.93087 | 115.41536 | Marine or coastal | 300 | 0 |
| Guangdong Nanpeng Archipelago | 23.28224 | 117.25586 | Marine or coastal | 69 | -45 |
| Hangzhou Xixi Wetlands | 30.26887 | 120.05993 | Inland | 10 | 3 |
| Heilongjiang Nanweng River | 51.32056 | 125.38111 | Inland | 1044 | 370 |
| Heilongjiang Qixing River | 46.73833 | 132.23139 | Inland | 59 | 50 |
| Heilongjiang Zhenbaodao Wetland | 46.12778 | 133.63722 | Inland | 60 | 224 |
| Honghe National Nature Reserve | 47.81667 | 133.66667 | Inland | 55 | 51 |
| Hubei Dajiu Lake Wetland | 31.47066 | 110.04748 | Inland | 2624 | 1550 |
| Jilin Momoge National Nature Reserve | 45.90889 | 123.76556 | Human-made | 161 | 128 |
| Lashihai Wetland | 26.89554 | 100.13772 | Inland | 3100 | 2441 |
| Mai Po Marshes and Inner Deep Bay | 22.48889 | 114.04556 | Marine or coastal | 0 | 0 |
| Mapangyong Cuo | 30.69410 | 81.38807 | Inland | 6500 | 4500 |
| Napahai Wetland | 27.85446 | 99.64578 | Inland | 3266 | 3266 |
| Niaodao | 36.83333 | 100.16667 | Inland | 3250 | 3185 |
| Poyanghu | 29.16667 | 115.98333 | Inland | 18 | 12 |
| Shandong Yellow River Delta Wetland | 37.76979 | 119.08561 | Marine or coastal | 5 | -3 |
| Shanghai Yangtze Estuarine Wetland | 31.51667 | 122.08333 | Marine or coastal | 0 | 0 |
| Shankou Mangrove Nature Reserve | 21.55542 | 109.68514 | Marine or coastal | 3 | 1 |
| Nan Dongting Wetland and Waterfowl | 28.83333 | 112.66667 | Human-made | 34 | 28 |
| Shuangtai Estuary | 40.91271 | 121.76145 | Inland | 4 | 0 |
| Xingkai Lake National Nature Reserve | 45.26616 | 132.66842 | Inland | 81 | 59 |
| Sichuan Ruoergai Wetland | 33.71667 | 102.81667 | Inland | 3704 | 3422 |
| Xi dongting lake nature reserve | 29.01667 | 112.08333 | Human-made | 59 | 20 |
| Yancheng National Nature Reserve | 33.51667 | 120.36667 | Human-made | 3 | 0 |
| Zhangye Heihe Wetland | 39.49893 | 99.76972 | Inland | 1500 | 1200 |
| Zhanjiang Mangrove | 20.90000 | 110.13333 | Marine or coastal | 3 | 0 |
| Chongming Dongtan, Shanghai | 31.48704 | 121.96237 | Human-made | 5 | 0 |
| Fujian Zhangjiangkou Mangrove | 23.91667 | 117.41667 | Marine or coastal | 8 | 0 |
| Shandong Jining Nansi Lake | 34.89445 | 116.95534 | Inland | 36 | 30 |
| Tianjin Beidagang Wetlands | 38.79431 | 117.35853 | Inland | 5 | 4 |
| Henan Minquan Yellow River Gudao | 34.65928 | 115.32231 | Inland | 66 | 59 |
| Heilongjiang Hadong Yanjiang | 45.92615 | 126.82127 | Inland | 139 | 107 |
| Tibet Trari Nam Co Wetlands | 30.94101 | 85.58295 | Inland | 4930 | 4608 |
| Inner Mongolia Bila River Wetlands | 49.44152 | 123.31735 | Inland | 886 | 377 |
| Jiangxi Poyang Lake Nanji Wetlands | 29.00530 | 116.29705 | Inland | 49 | 8 |
| Maidika | 31.01683 | 92.84866 | Inland | 5000 | 4800 |
| Gansu Yellow River Shouqu Wetlands | 33.57742 | 102.18457 | Inland | 4060 | 3420 |

**References**

1. Ramsar. The Ramsar Sites Criteria: The nine criteria for identifying Wetlands of International Importance. *ramsar.org* https://www.ramsar.org/sites/default/files/documents/library/ramsarsites_criteria_eng.pdf (2022).

2. DCCEEW. Criteria for identifying Wetlands of International Importance. *Department of Climate Change, Energy, the Environment and Water* https://www.dcceew.gov.au/water/wetlands/ramsar/criteria-identifying-wetlands (2022).
